# Supplementary material for: Cancer discrimination by on-cell N-glycan ligation
Source: Commun Chem. 2020 Feb 26;3:26. doi: 10.1038/s42004-020-0270-9 (PMC9814842; doi:10.1038/s42004-020-0270-9)
Supplement: Supplementary file 1 — Supplementary Information [file 42004_2020_270_MOESM1_ESM.pdf]

Supplementary Information

Cancer discrimination by on-cell N-glycan ligation

Nomura, S., Tanaka, K. et al.

Supplementary Methods

1. Chemical Syntheses and Analysis

1.1 General Information

N-Glycan derivatives were supplied from GlyTech Inc. (Kyoto, Japan). Boc-miniPEG™, Boc-miniPEG-3™, and EDC·HCl were obtained from Peptide Institute, Inc. (Osaka, Japan). 5-(and-6)-Carboxytetramethylrhodamine, succinimidyl ester (5(6)-TAMRA, SE) was obtained from AAT Bioquest (California, USA). 3-(5-Carboxypentyl)-1,1-dimethyl-2-((*E*)-2-((*E*)-3((*E*)-2-(1,1,3-trimethyl-1*H*-benzo[e]indol-2(3*H*)-ylidene)ethylidene)cyclohex-1-enyl)vinyl)-1*H*-benzo[e]indolium (Cy7.5) chloride was obtained from abcam (Cambridge, UK). All other chemicals and solvents of special grade were obtained from Tokyo Chemical Industry, co., Ltd (Tokyo, Japan) or Wako Pure Chemical Industries, Ltd (Osaka, Japan), and were used without purification. HPLC was performed on Shimadzu liquid chromatograph CBM-20A, LC-20AD, and SPD-20AV (Kyoto, Japan) with an analytical column COSMOSIL $_5C_{18}$-AR-300 (4.6 mm x 250 mm, Nacalai Tesque, Inc., Kyoto, Japan) at a flow rate of 1 mL/min, and on JASCO liquid chromatograph LC-NetII/ADC, PU-2089 Plus, and UV-2075 Plus (Tokyo, Japan) with a preparative column COSMOSIL $_5C_{18}$-AR-300 (20 mm x 250 mm, Nacalai Tesque, Inc.) at a flow rate of 7 mL/min. Mass spectra were recorded on a Bruker micrOTOF QIII (Rheinstetten, Germany). NMR spectra were recorded on a JEOL AL400 spectrometer (Tokyo, Japan).

1.2 Preparation of N-Glycan Ligands

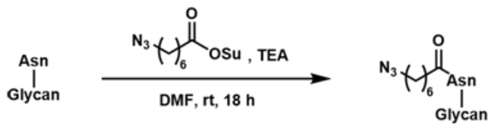

4a-e **5a-e**

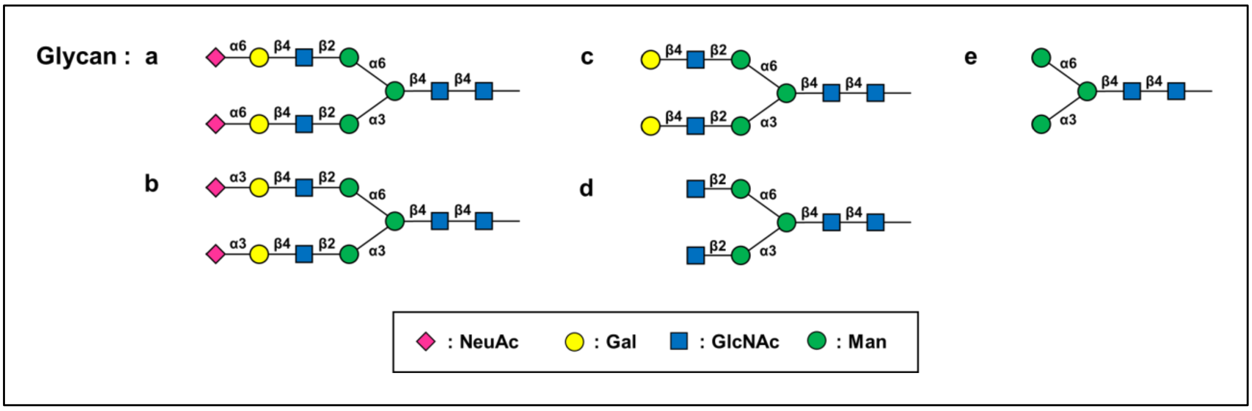

Preparation of **5a** and **5c**.

The starting glycans **4a-e** were prepared by reported procedure.[ref 1]

Glycan derivatives **5a** and **5c** were prepared as reported previously.[ref 2,3]

Glycan derivative **5a**: HRMS calcd for $C_{95}H_{154}N_{11}O_{65}$ ([M−H]$^{-}$) 2488.9089, found 2488.9119. t_R: 15.4 min (flow rate of 1 mL/min with a linear gradient from 1-60% MeCN/0.1% TFA for 30 min using the analytical column).

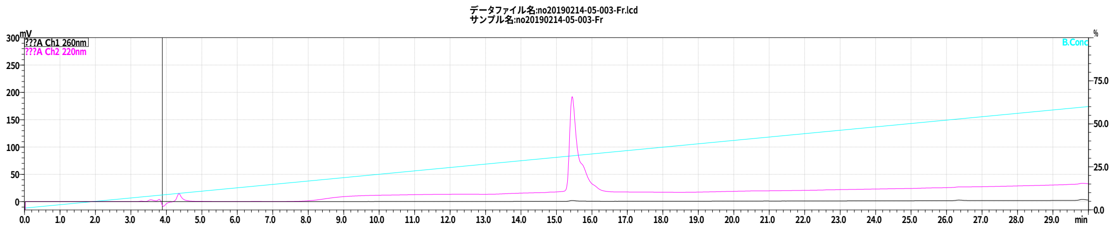

Supplementary Figure 1. HPLC chromatogram of **5a**.

Glycan derivative **5c**: HRMS calcd for $C_{73}H_{120}N_9O_{49}$ ([M−H]$^{-}$) 1906.7180, found 1906.7180. t_R: 16.0 min (flow rate of 1 mL/min with a linear gradient from 1-60% MeCN/0.1% TFA for 30 min using the analytical column).

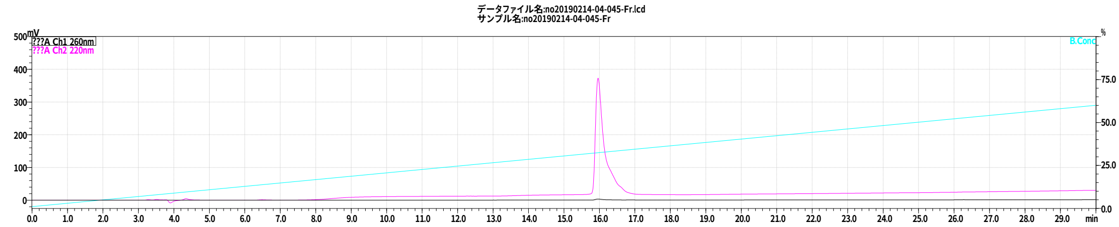

Supplementary Figure 2. HPLC chromatogram of **5c**.

Preparation of **5b**.

To a solution of **4b** (23.4 mg, 10 μmol) and 7-azidoheptanoic acid succinimidyl ester (8.1 mg, 30 μmol) in DMF (1.5 mL) was added Et_3N (4.2 μL, 30 μmol) and the mixture was stirred overnight at room temperature for 18 h. After the solvent was diluted with H_2O and filtrated, the solution was purified by RP-HPLC using the preparative column at a flow rate of 7 mL/min with a linear gradient of MeCN/0.1% TFA (1-25% for 80 min, t_R: 47-50 min) to yield **5b** (21.2 mg, 85%). HRMS calcd for $C_{95}H_{154}N_{11}O_{65}$ ($[M-H]^-$) 2488.9089, found 2488.9129. t_R: 15.4 min (flow rate of 1 mL/min with a linear gradient from 1-60% MeCN/0.1% TFA for 30 min using the analytical column).

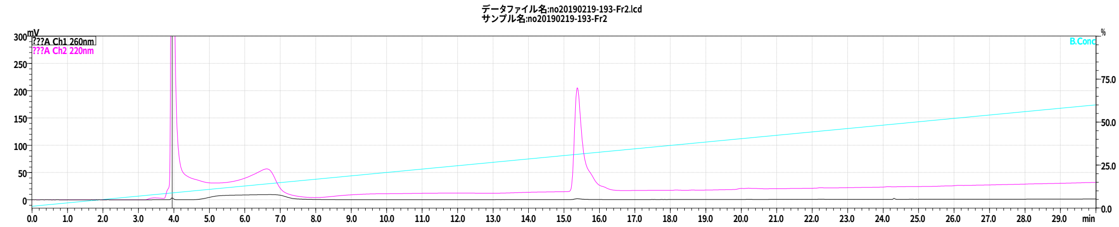

Supplementary Figure 3. HPLC chromatogram of **5b**.

Preparation of **5d**.

To a solution of **4d** (14.3 mg, 10 μmol) and 7-azidoheptanoic acid succinimidyl ester (8.1 mg, 30 μmol) in DMF (1.0 mL) was added Et_3N (4.2 μL, 30 μmol) and the mixture was stirred overnight at room temperature for 18 h. After the solvent was diluted with H_2O and filtrated, the solution was purified by RP-HPLC using the preparative column at a flow rate of 7 mL/min with a linear gradient of MeCN/0.1% TFA (1-25% for 80 min, t_R: 54-58 min) to yield **5d** (15.7 mg, 99%). HRMS calcd for $C_{61}H_{100}N_9O_{39}$ ($[M-H]^-$) 1582.6124, found 1582.6158. t_R: 16.3 min (flow rate of 1 mL/min with a linear gradient from 1-60% MeCN/0.1% TFA for 30 min using the analytical column).

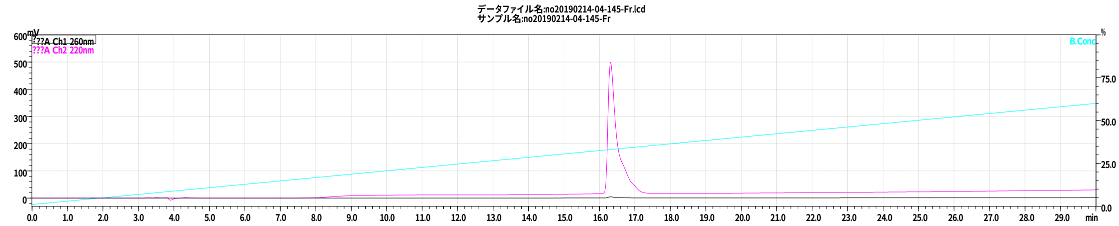

Supplementary Figure 4. HPLC chromatogram of **5d**.

Preparation of **5e**.

To a solution of **4e** (10.2 mg, 10 μmol) and 7-azidoheptanoic acid succinimidyl ester (8.1 mg, 30 μmol) in DMF (1.0 mL) was added Et_3N (4.2 μL, 30 μmol) and the mixture was stirred overnight at room temperature for 18 h. After the solvent was diluted with H_2O and filtrated, the solution was purified by RP-HPLC using the preparative column at a flow rate of 7 mL/min with a linear gradient of MeCN/0.1% TFA (1-25% for 80 min, t_R: 52-56 min) to yield **5e** (11.6 mg, 98%). HRMS calcd for $C_{45}H_{74}N_7O_{29}$ ([M−H]$^-$) 1176.4536, found 1176.4546. t_R: 16.6 min (flow rate of 1 mL/min with a linear gradient from 1-60% MeCN/0.1% TFA for 30 min using the analytical column).

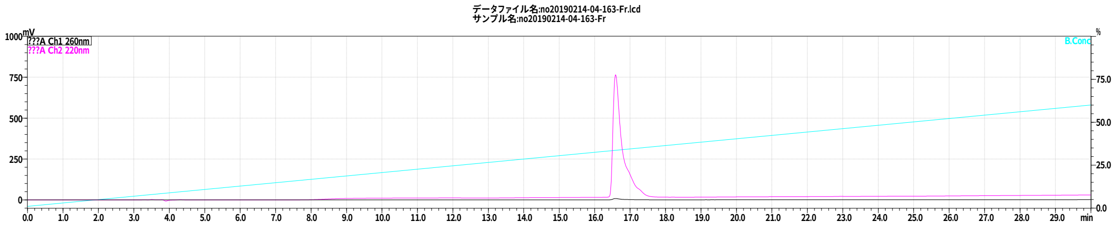

Supplementary Figure 5. HPLC chromatogram of **5e**.

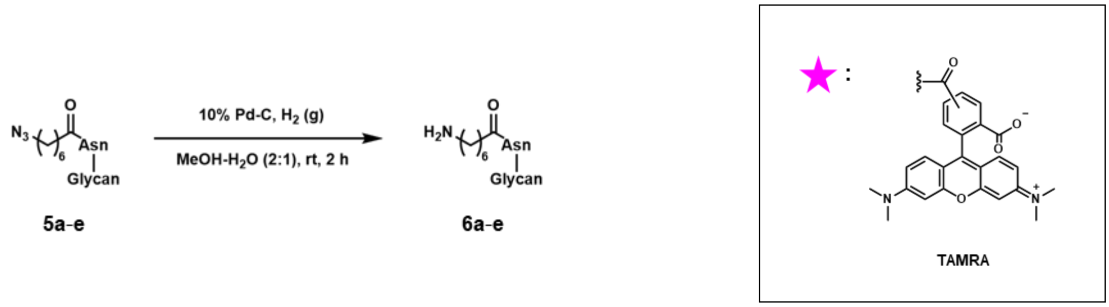

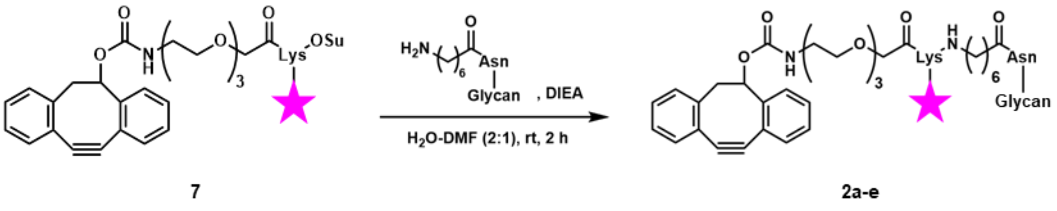

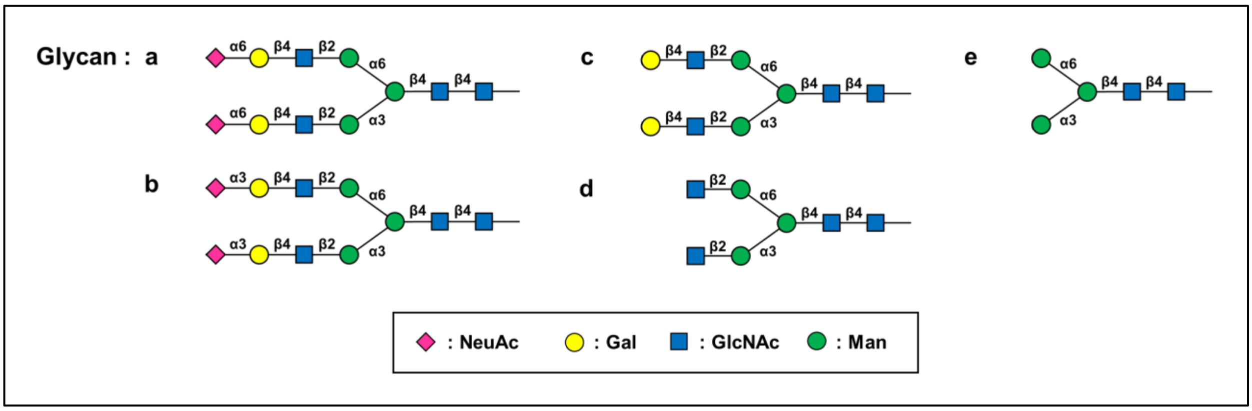

Preparation of **2a** and **2c**.

Intermediate **7** and glycan ligands **2a** and **2c** were prepared as reported previously.[ref 2, 3]

Glycan ligand **2a**: HRMS calcd for $C_{151}H_{213}N_{14}O_{76}$ ($[M-H]^-$) 3438.3238, found 3438.3147. t_R: 18.2 and 19.6 min for mixture of TAMRA regioisomers (flow rate of 1 mL/min with a linear gradient from 20-80% MeCN/0.1% TFA for 30 min using the analytical column).

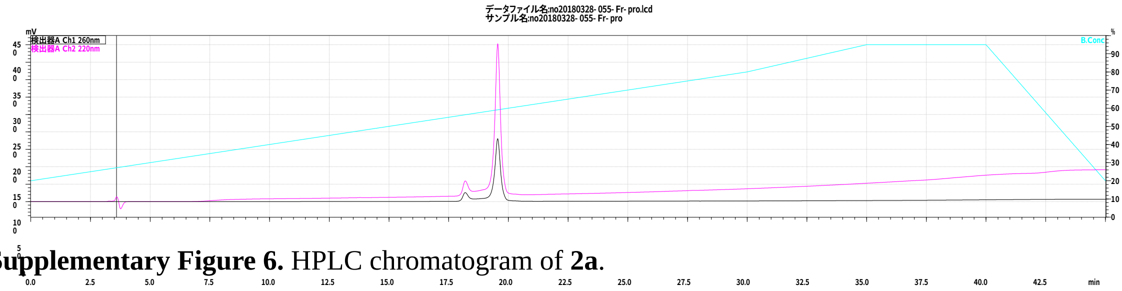

Supplementary Figure 6. HPLC chromatogram of **2a**.

Glycan ligand **2c**: HRMS calcd for $C_{129}H_{179}N_{12}O_{60}$ ($[M-H]^-$) 2856.1330, found 2856.1282. t_R: 18.6 and 20.0 min for mixture of TAMRA regioisomers (flow rate of 1 mL/min with a linear gradient from 20-80% MeCN/0.1% TFA for 30 min using the analytical column).

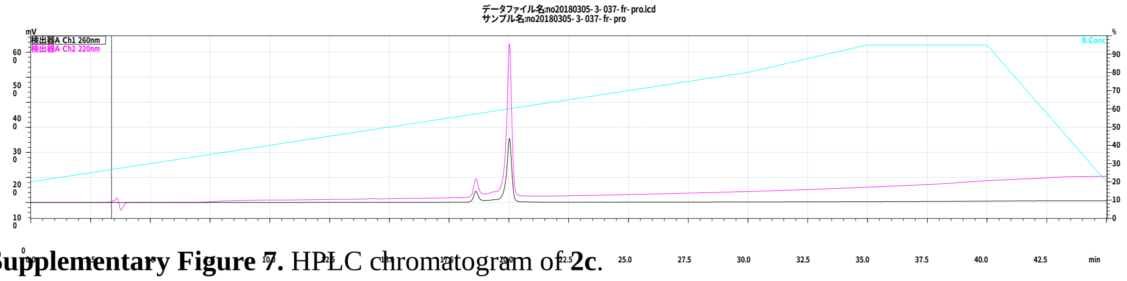

Supplementary Figure 7. HPLC chromatogram of **2c**.

Preparation of **2b**.

To a solution of **5b** (10.0 mg, 4.0 μmol) in MeOH/H_2O (2:1, v/v, 4.0 mL) was added 10% palladium on carbon (with 50% H_2O, 28.4 mg, 12 μmol). After the reaction mixture was stirred under H_2 atmosphere for 3 h, the catalyst was removed by filtration, and concentration *in vacuo*. The residue was dissolved in H_2O and lyophilized to yield the amine **6b** (8.2 mg, 83%), which was rapidly analyzed by HRMS and immediately used for consideration with **7** without further purification. HRMS calcd for $C_{95}H_{156}N_9O_{65}$ ($[M-H]^-$) 2462.9184, found 2462.9184.

To a solution of **7**[ref 3] (1.6 mg, 1.5 μmol) in DMF (100 μL), was added a solution of **6b** obtain above (2.5 mg, 1.0 μmol) in H_2O (200 μL) and DIEA (1.2 μL, 7.0 μmol). After the reaction mixture was stirred for 2 h at room temperature, the mixture was quenched with AcOH (50 μL), and directly purified by RP-HPLC using the preparative column at a flow rate of 7 mL/min with a linear gradient of MeCN/0.1% TFA (40-55% for 80 min, t_R: 35-36 and 41-43 min for mixture of TAMRA regioisomers) to yield **2b** (3.1 mg, 89%) as a mixture of TAMRA regioisomers. HRMS calcd for $C_{151}H_{213}N_{14}O_{76}$ ($[M-H]^-$) 3438.3238, found 3438.3232. t_R: 18.3 and 19.6 min for mixture of TAMRA regioisomers (flow rate of 1 mL/min with a linear gradient from 20-80% MeCN/0.1% TFA for 30 min using the analytical column).

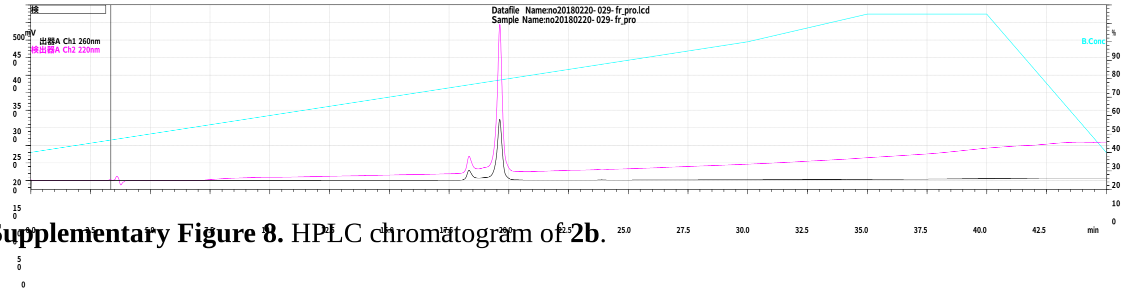

Supplementary Figure 8. HPLC chromatogram of **2b**.

Preparation of **2d**.

To a solution of **5d** (14.3 mg, 9.0 μmol) in MeOH/H_2O (2:1, v/v, 1.0 mL) was added 10% palladium on carbon (with 50% H_2O, 63.9 mg, 27 μmol). After the reaction mixture was stirred under H_2 atmosphere for 4 h, the catalyst was removed by filtration, and concentration *in vacuo*. The residue was dissolved in H_2O and lyophilized to obtain the amine **6d** (13.3 mg, 95%), which was rapidly analyzed by HRMS and immediately used for consideration with **6d** without further purification. HRMS calcd for $C_{61}H_{102}N_7O_{39}$ ([M−H]$^-$) 1556.6219, found 1556.6243.

To a solution of **7**[ref 3] (4.9 mg, 4.50 μmol) in DMF (200 μL), was added a solution of **6d** obtain above (4.7 mg, 3.0 μmol) in H_2O (400 μL) and DIEA (3.7 μL, 21 μmol). After the reaction mixture was stirred for 2 h at room temperature, the mixture was quenched with AcOH (50 μL), and directly purified by RP-HPLC using the preparative column at a flow rate of 7 mL/min with a linear gradient of MeCN/0.1% TFA (40-55% for 80 min, t_R: 34-36 and 40-43 min for mixture of TAMRA regioisomers) to yield **2d** (7.4 mg, 97%) as a mixture of TAMRA regioisomers. HRMS calcd for $C_{117}H_{159}N_{12}O_{50}$ ([M−H]$^-$) 2532.0273, found 2532.0272. t_R: 18.7 and 20.1 min for mixture of TAMRA regioisomers (flow rate of 1 mL/min with a linear gradient from 20-80% MeCN/0.1% TFA for 30 min using the analytical column).

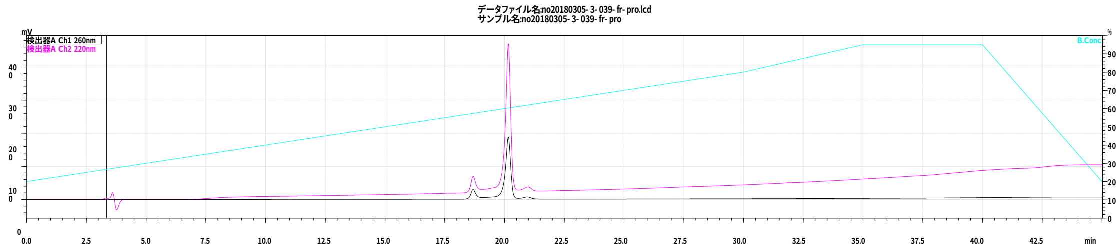

Supplementary Figure 9. HPLC chromatogram of **2d**.

Preparation of **2e**.

To a solution of **5e** (11.2 mg, 9.5 μmol) in MeOH/H_2O (2:1, v/v, 9.5 mL) was added 10% palladium on carbon (with 50% H_2O, 67.4 mg, 28.5 μmol). After the reaction mixture was stirred under H_2 atmosphere for 2 h, the catalyst was removed by filtration, and concentration *in vacuo*. The residue was dissolved in H_2O and lyophilized to obtain the amine **6e** (9.8 mg, 90%), which was rapidly analyzed by HRMS and immediately used for consideration with **7** without further purification. HRMS calcd for $C_{45}H_{76}N_5O_{29}$ ([M−H]$^-$) 1150.4631, found 1150.4725.

To a solution of **7**[ref 3] (3.3 mg, 3.0 μmol) in DMF (100 μL), was added a solution of **6e** obtain above (2.3 mg, 2.0 μmol) in H_2O (200 μL) and DIEA (2.4 μL, 14 μmol). After the reaction mixture was stirred for 2 h at room temperature, the mixture was quenched with AcOH, and directly purified by RP-HPLC using the preparative column at a flow rate of 7 mL/min with a linear gradient of MeCN/0.1% TFA (40-55% for 80 min, t_R: 40-42 and 47-50 min for mixture of TAMRA regioisomers) to yield **2e** (3.8 mg, 90%) as a mixture of TAMRA regioisomers. HRMS calcd

for $C_{101}H_{133}N_{10}O_{40}$ ([M−H]$^-$) 2125.8686, found 2125.8618. t_R: 19.0 and 20.6 min for mixture of TAMRA regioisomers (flow rate of 1 mL/min with a linear gradient from 20-80% MeCN/0.1% TFA for 30 min using the analytical column).

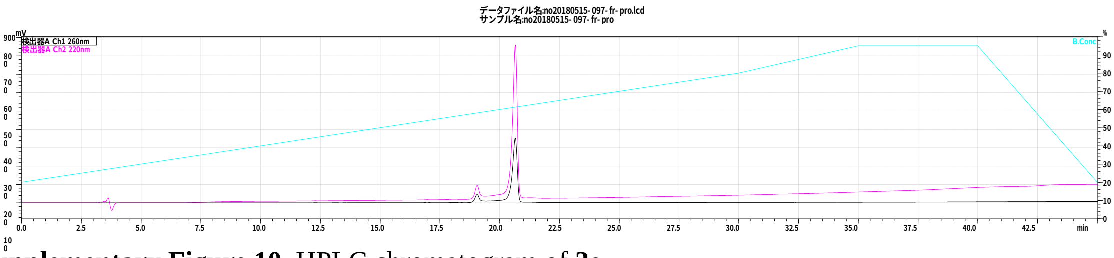

Supplementary Figure 10. HPLC chromatogram of **2e**.

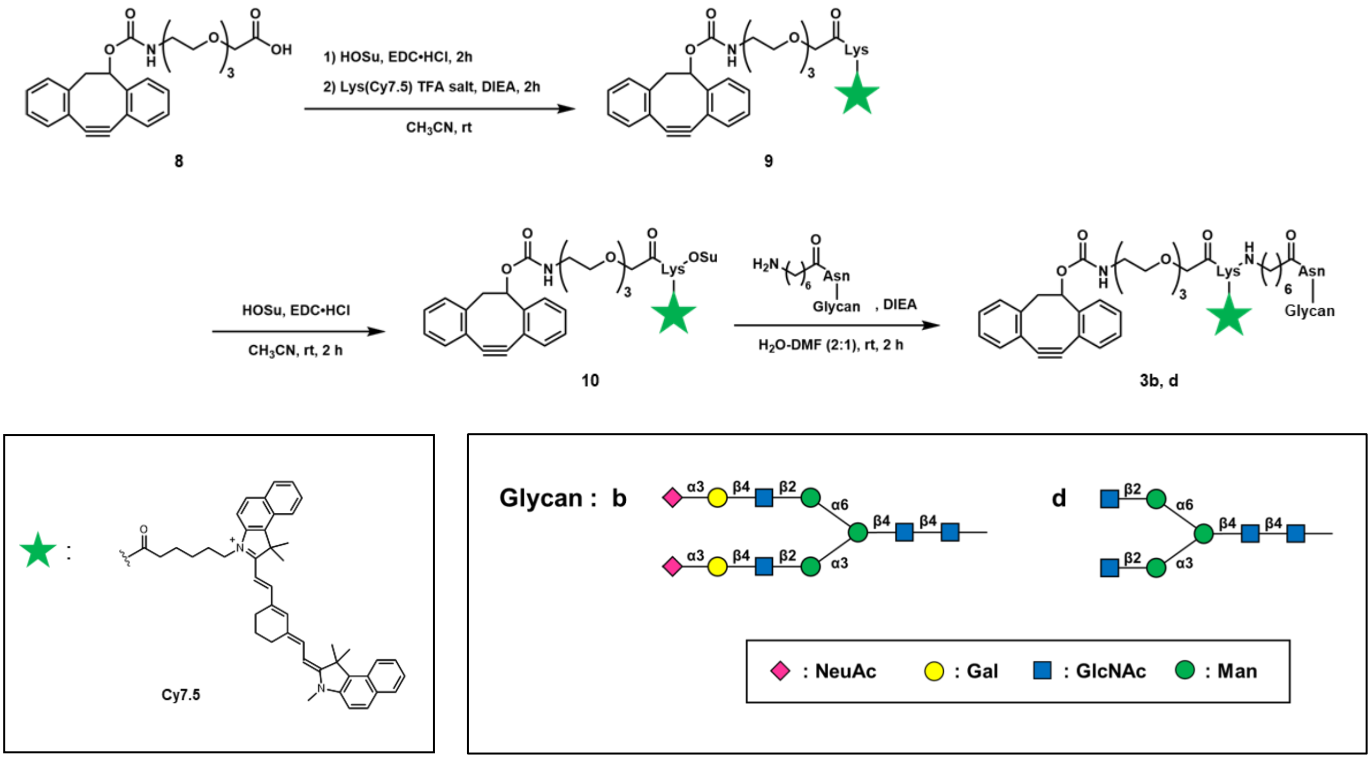

Labeling of lysine ε-amino group by Cy7.5 (Lys(Cy7.5) • TFA salt).

To a solution of Boc-Lys (4.1 mg, 16.5 μmol) in 10% H_2O-MeCN solution (300 μL) was added Cy7.5 chloride (11.7 mg, 15 μmol) and DIEA (7.8 μL, 45 μmol). The reaction mixture was stirred for 3 h at room temperature, quenched by AcOH (50 μL), and purified by RP-HPLC using the preparative column at a flow rate of 7 mL/min with a linear gradient of MeCN/0.1% TFA (60-75% for 80 min, t_R: 43-49 min) to yield Boc-Lys(Cy7.5). Boc-Lys(Cy7.5) thus obtained was dissolved in TFA (5.0 mL) and the reaction mixture was stirred for 10 min at room temperature. After removal of excess TFA with N_2 gas, the resultant residue was dissolved in H_2O, and lyophilized to yield Lys(Cy7.5) • TFA salt (12.1 mg, 99%). HRMS calcd for $C_{51}H_{61}N_4O_3$ ($[M]^+$) 777.4738, found 777.4733. t_R: 15.1 min (flow rate of 1 mL/min with a linear gradient from 50 to 95% MeCN/0.1% TFA for 30 min using the analytical column).

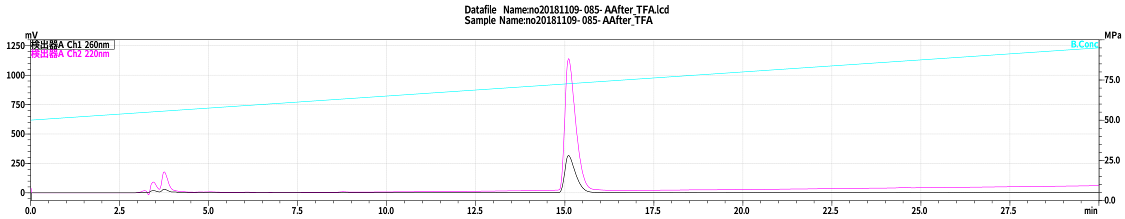

Supplementary Figure 11. HPLC chromatogram of Lys(Cy7.5) • TFA salt.

Preparation of **9**.

To a solution of **8**[ref 3] (1.45 mg, 3.20 μmol) in 10% H_2O-MeCN solution (200 μL) was added *N*-hydroxysuccinimide (0.6 mg, 4.8 μmol) and EDC • HCl (0.9 mg, 4.8 μmol). After the reaction mixture was stirred for 2 h at room temperature, Lys(Cy7.5) • TFA salt prepared above (3.5 mg, 3.5 μmol) and DIEA (2.8 μL, 16 μmol) were further added to this mixture and stirred for additional 2 h. The resulting mixture was quenched with AcOH (50 μL), and concentrated *in vacuo*. The residue was purified by RP-HPLC using the preparative column at a flow rate of 7 mL/min with a linear gradient of MeCN / 0.1% TFA (65-80% for 80 min, t_R: 45-50 min) to yield **9** (4.0 mg, 94%). HRMS calcd for $C_{76}H_{86}N_5O_9$ ($[M]^+$) 1212.6420, found 1212.6352. t_R: 28.2 min (flow rate of 1 mL/min with a linear gradient from 50 to 95% MeCN/0.1% TFA for 30 min using the analytical column).

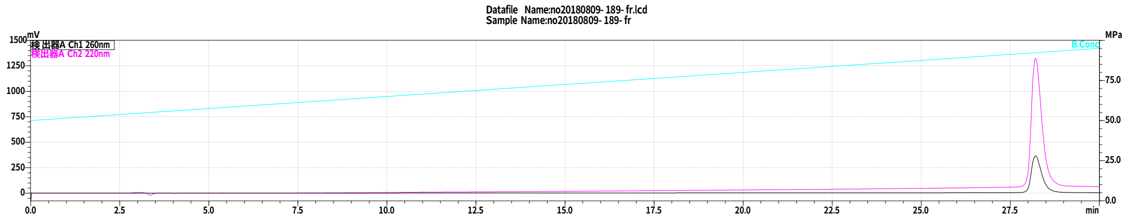

Supplementary Figure 12. HPLC chromatogram of **9**.

Preparation of **10**.

To a solution of **9** obtained above (6.6 mg, 5.0 μmol) in MeCN (500 μL) was added *N*-hydroxysuccinimide (2.9 mg, 25 μmol) and EDC • HCl (4.8 mg, 25 μmol). The solution was stirred for 2 h at room temperature, and the mixture was directly purified by RP-HPLC using the preparative column at a flow rate of 7 mL/min with a linear gradient of MeCN / 0.1% TFA (65-80% for 80 min, t_R: 35-38 min) to yield **10** (5.7 mg, 81%). HRMS calcd for $C_{80}H_{89}N_6O_{11}$ ($[M]^+$) 1309.6584, found 1309.6611. t_R: 29.4 min (flow rate of 1 mL/min with a linear gradient from 50 to 95% MeCN/0.1% TFA for 30 min using the analytical column).

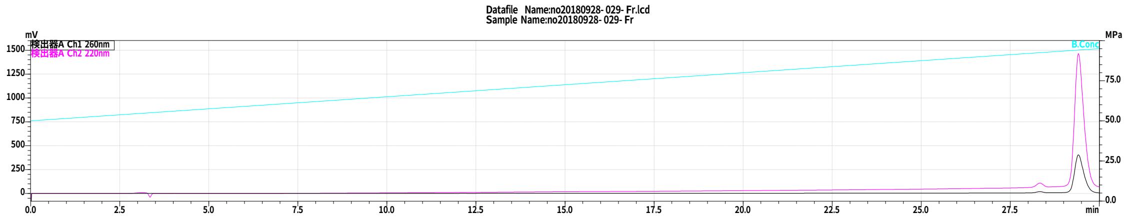

Supplementary Figure 13. HPLC chromatogram of **10**.

Preparation of **3b**.

To a solution of **10** (4.3 g, 3.0 µmol) in DMF (100 µL) was added a solution of **6b** (4.9 mg, 2.0 µmol) in DMF/H_2O (1:1, v/v, 100 µL) and DIEA (1.6 µL, 9.0 µmol). The reaction mixture was stirred for 2 h at room temperature, quenched with AcOH (50 µL), and purified by RP-HPLC using the preparative column at a flow rate of 7 mL/min with a linear gradient of MeCN/0.1% TFA (50-80% for 80 min, t_R: 42-46 min) to yield **3b** (2.1 mg, 29%). HRMS calcd for $C_{171}H_{241}N_{14}O_{73}$ ($[M]^+$) 3658.5571, found 3658.5556. t_R: 16.9 min (flow rate of 1 mL/min with a linear gradient from 50 to 95% MeCN/0.1% TFA for 30 min using the analytical column).

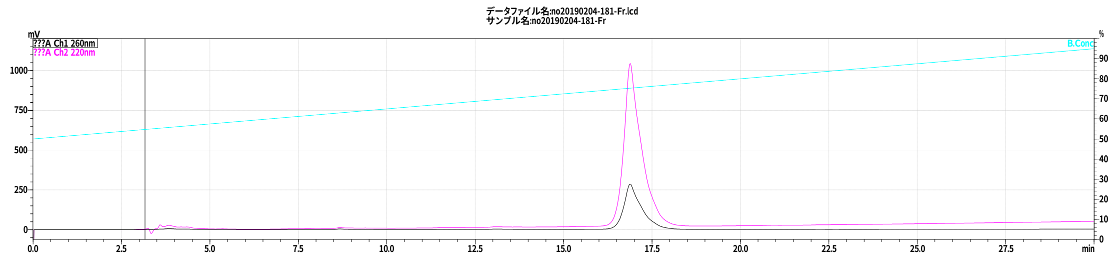

Supplementary Figure 14. HPLC chromatogram of **3b**.

Preparation of **3d**.

To a solution of **10** (5.7 mg, 4.0 µmol) in DMF (100 µL) was added a solution of **6d** (3.1 mg, 2.0 µmol) in DMF/H_2O (1: 1, v/v, 100 µL) and DIEA (2.1 µL, 12 µmol). The reaction mixture was stirred for 2 h at room temperature, quenched with AcOH (50 µL), and purified by RP-HPLC using the preparative column at a flow rate of 7 mL/min with a linear gradient of MeCN/0.1% TFA (50-80% for 80 min, t_R: 45-50 min) to yield **3d** (1.9 mg, 34%). HRMS calcd for $C_{137}H_{187}N_{12}O_{47}$ ($[M]^+$) 2752.2606, found 2752.2768. t_R: 18.1 min (flow rate of 1 mL/min with a linear gradient from 50 to 95% MeCN/0.1% TFA for 30 min using the analytical column).

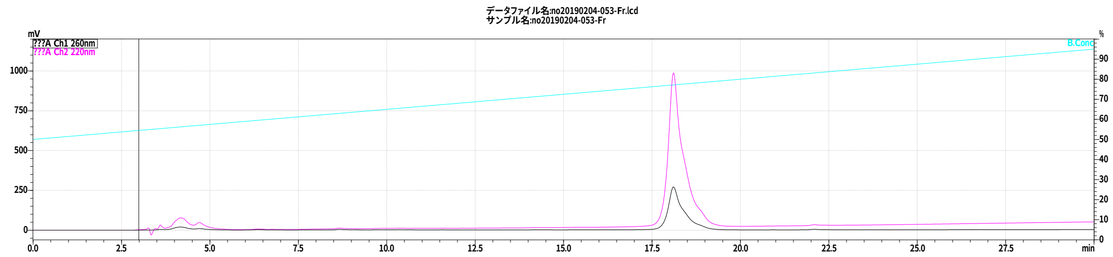

Supplementary Figure 15. HPLC chromatogram of **3d**.

2. Cell-based Experiments

2.1 General Information

The six cell lines used in this study were obtained from either American Type Culture Collection (Virginia, USA), RIKEN Cell Bank, or JCRB Cell Bank. HeLaS3, A549, BxPC3, PC3 and TIG3 cells were cultured in DMEM (Wako) containing 10% fetal bovine serum (FBS, Wako) and penicillin-streptomycin (Thermo Fisher Scientific, Inc., Massachusetts, USA). SW620 cells were cultured in Leibovitz's L-15 Medium (Wako) containing 10% FBS and penicillin-streptomycin. We check the mycoplasma contamination regularly and also before performing this research.

2.2 *In Vitro* Imaging Studies

HeLaS3, A549, BxPC3, PC3, TIG3 and SW620 cells were seeded onto 96-well plates. A solution of the cyclic RGDyK peptide ligands **1a-d** (50 µM, 100 µL) in medium was added to the cells, and the solution was incubated for 15 min at room temperature. As a positive control, a solution containing only TAMRA-labeled cyclic RGDyK peptide[ref 3] was also incubated at same concentration. After washing the cells twice with medium, the cells were treated with a solution of glycan ligands **2a-2e** (100 µM, 25 µL) in medium for 30 min at 4°C. The cells were then washed with medium, fixed with 4% paraformaldehyde in PBS for 10 min, and labeled with Hoechest33342 (Thermo Fisher Scientific, Inc.) to count cell number. The samples were imaged using an BZ-X 700 (Keyence, Osaka, Japan) or FV3000 (Olympus, Tokyo, Japan). Fluorescent intensity was calculated by ImageJ (U. S. National Institutes of Health, Maryland, USA), and the scores were normalized by cell number. The labeling experiments on the plate were performed for four times each cell lines, and data was averaged.

For example, we performed the imaging of A549 cells by treating with **2b** or **2e** at the elevated temperature, instead of performing the click reaction at 4°C. We still cannot see any fluorescence signal by the treatment with **2e**, but in addition, the fluorescence intensity was significantly reduced even for the case of **2b** (Fig. S16). As analyzed by confocal microscopy, the treatment of A549 cells with TAMRA-labeled cyclic RGDyK at RT notably accelerated the internalization and accumulated in the nuclei (Fig. S17). These images clearly showed that the higher rate of integrin-mediated internalization of the on-cell cyclic RGDyK reduced the efficiency of "on-cell" click reaction.

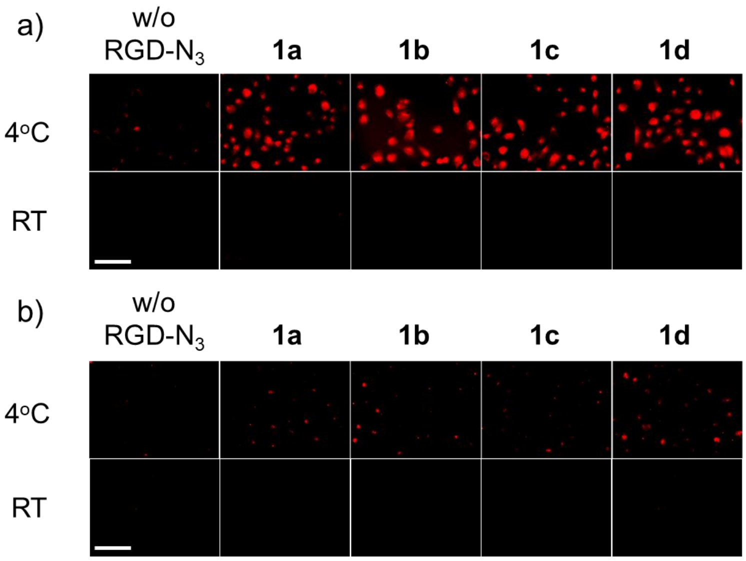

Supplementary Figure 16. The A549 cells were treated with cyclic RGDyK ligands **1a-d** (50 μM, RT, 15 min) and then fluorescently labeled N-glycan ligands, a) **2b** or b) **2e** (100 μM, 4°C or RT, 30 min). Scale bar, 100 μm.

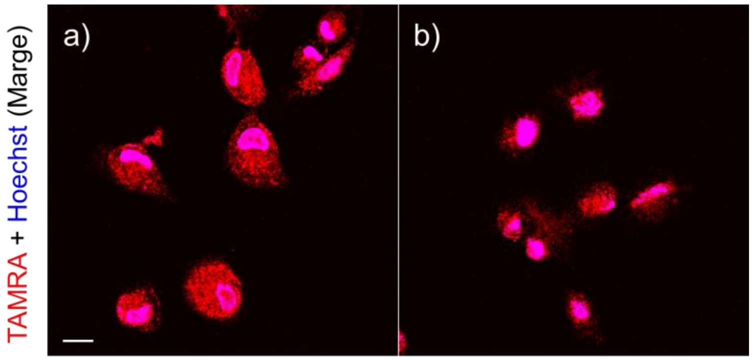

Supplementary Figure 17. Confocal microscopy images of A549: The cells were treated with TAMRA-cyclic RGDyK peptide (50 μM, RT, 15 min), washed, and then incubated at a) 4°C or b) RT for 30 min. Scale bar, 20 μm.

Supplementary Table 1. Average fluorescent intensity in Fig. 3 (n [plate =4] and normalized by cell number).

a) HeLaS3

Glycan ligands	w/o RGD-N_3	**1a**	**1b**	**1c**	**1d**
**2a**	0.001 ± 0.002	0.005 ± 0.004	0.005 ± 0.005	0.005 ± 0.008	0.004 ± 0.004
**2b**	0.038 ± 0.044	0.019 ± 0.024	0.001 ± 0.002	0.020 ± 0.011	0.003 ± 0.003
**2c**	0.010 ± 0.007	0.028 ± 0.017	0.010 ± 0.008	0.004 ± 0.007	0.011 ± 0.011
**2d**	0.084 ± 0.058	0.227 ± 0.134	0.133 ± 0.109	0.490 ± 0.223	0.521 ± 0.265
**2e**	0.009 ± 0.008	0.132 ± 0.123	0.231 ± 0.113	0.351 ± 0.148	0.368 ± 0.179

b) A549

Glycan ligands	w/o RGD-N_3	**1a**	**1b**	**1c**	**1d**
**2a**	0.030 ± 0.009	0.143 ± 0.043	0.061 ± 0.033	0.095 ± 0.052	0.000 ± 0.000
**2b**	0.075 ± 0.029	0.555 ± 0.163	1.504 ± 0.066	0.789 ± 0.126	0.998 ± 0.143
**2c**	0.013 ± 0.004	0.092 ± 0.065	0.156 ± 0.032	0.210 ± 0.081	0.086 ± 0.056
**2d**	0.009 ± 0.008	0.011 ± 0.012	0.006 ± 0.006	0.013 ± 0.017	0.026 ± 0.021
**2e**	0.007 ± 0.005	0.032 ± 0.010	0.049 ± 0.033	0.024 ± 0.006	0.141 ± 0.060

c) BxPC3

Glycan ligands	w/o RGD-N_3	**1a**	**1b**	**1c**	**1d**
**2a**	0.019 ± 0.017	0.006 ± 0.004	0.010 ± 0.008	0.001 ± 0.001	0.017 ± 0.023
**2b**	0.021 ± 0.022	0.057 ± 0.060	0.006 ± 0.006	0.003 ± 0.001	0.005 ± 0.008
**2c**	0.011 ± 0.006	0.369 ± 0.231	0.138 ± 0.144	0.078 ± 0.052	0.025 ± 0.011
**2d**	0.053 ± 0.012	0.020 ± 0.017	0.158 ± 0.081	0.210 ± 0.154	0.342 ± 0.231
**2e**	0.122 ± 0.079	0.107 ± 0.064	0.072 ± 0.038	1.116 ± 0.174	0.121 ± 0.107

d) PC3

Glycan ligands	w/o RGD-N_3	**1a**	**1b**	**1c**	**1d**
**2a**	0.014 ± 0.016	0.022 ± 0.016	0.029 ± 0.009	0.027 ± 0.017	0.055 ± 0.042
**2b**	0.056 ± 0.046	0.047 ± 0.011	0.030 ± 0.012	0.058 ± 0.019	0.044 ± 0.018
**2c**	0.239 ± 0.090	0.283 ± 0.272	0.322 ± 0.190	0.244 ± 0.091	0.314 ± 0.151
**2d**	0.052 ± 0.012	0.032 ± 0.018	0.073 ± 0.022	0.130 ± 0.120	0.083 ± 0.058
**2e**	0.044 ± 0.044	0.057 ± 0.030	0.055 ± 0.028	0.068 ± 0.051	0.060 ± 0.055

e) SW620

Glycan ligands	w/o RGD-N_3	**1a**	**1b**	**1c**	**1d**
**2a**	0.073 ± 0.074	0.057 ± 0.014	0.138 ± 0.086	0.384 ± 0.212	0.426 ± 0.269
**2b**	0.028 ± 0.037	0.292 ± 0.229	0.653 ± 0.400	0.860 ± 0.283	1.015 ± 0.308
**2c**	0.015 ± 0.009	0.005 ± 0.006	0.016 ± 0.014	0.001 ± 0.001	0.006 ± 0.006
**2d**	0.066 ± 0.081	0.060 ± 0.072	0.203 ± 0.192	0.322 ± 0.096	0.397 ± 0.138
**2e**	0.008 ± 0.007	0.002 ± 0.002	0.001 ± 0.001	0.025 ± 0.025	0.046 ± 0.044

f) TIG3

Glycan ligands	w/o RGD-N_3	**1a**	**1b**	**1c**	**1d**
**2a**	0.100 ± 0.079	0.061 ± 0.036	0.530 ± 0.156	0.605 ± 0.352	0.320 ± 0.220
**2b**	0.041 ± 0.028	0.266 ± 0.205	0.641 ± 0.300	0.218 ± 0.101	0.441 ± 0.274
**2c**	0.039 ± 0.035	0.011 ± 0.015	0.083 ± 0.035	0.036 ± 0.028	0.039 ± 0.035
**2d**	0.014 ± 0.012	0.118 ± 0.114	0.039 ± 0.025	0.227 ± 0.133	0.068 ± 0.061
**2e**	0.019 ± 0.013	0.005 ± 0.003	0.116 ± 0.069	0.242 ± 0.109	0.056 ± 0.041

Supplementary Table 2. Literature and Database survey of lectins expression levels in cancerous cells. a) Literature examples extracted from Table 1. mRNA-based lectin expression level analysis of HeLa, A549 and PC3 by b) Protein Atlas and c) RefEx data base.

a)

	2,6-Sia		2,3-Sia	Gal	GlcNAc	Man
HeLa	Siglec-3	—	Galectin-1	—	Vimentin	SP-D
A549	—	Siglec10	Galectin-1	—	—	SP-D
PC3	—	—	Galectin-1	—	Vimentin	—

b) Protein Atlas (https://www.proteinatlas.org/) /NX

	Siglec-3	Siglec-10	Galectin-1	Vimentin	SP-D
HeLa	0.2	0.2	1054.9	381.7	0
A549	0.1	0	1367.6	1009.9	0
PC3	23.9	0.5	3277.3	1292	0.2

c) RefEx (https://www.proteinatlas.org/)

	Siglec-3	Siglec-10	Galectin-1	Vimentin	SP-D
HaLa	2.68	0	5.27	6.12	0
A549	0	0	5.88	6.62	0.16
PC3	0.29	0	6.81	6.11	0

3. Animal Experiments

3.1 General Information

BALB/c-nu/nu mice (from 8 to 10 weeks old) were purchased from CLEA Japan (Tokyo, Japan). The mice were housed at the RIKEN Center for Biosystems Dynamics Research (BDR). All injections to the mice were performed under anesthesia. All procedures involving mouse experiments were approved by the Ethics Committee of RIKEN (MAH21-19-17), and performed in accordance with the institutional and national guidelines.

3.2 Selective Cancer Pre-targeting *In Vivo*

The cancer model mice were prepared by subcutaneously injecting HeLaS3 (2×10^6 cells/100 µL) or A549 (4×10^6 cells/100 µL) into the left shoulder of eight-ten weeks old BALB/cAJc1-nu/nu mice. The mice injected HeLaS3 or A549, were housed for 3 weeks or 4 weeks, respectively. The solution of cyclic RGDyK peptide **1d** (150 nmol/100 µL saline) was injected into mice via tail vein. After 30 min, the solutions of glycan ligands **3b** or **3d** (15 nmol/100 µL saline) were injected. After 3 h, the mice were dissected, and the fluorescence of cancer region was analyzed on an IVIS Spectrum *in vivo* imaging system (Caliper Life Sciences Inc, Massachusetts, USA, n=4).

a) HeLaS3

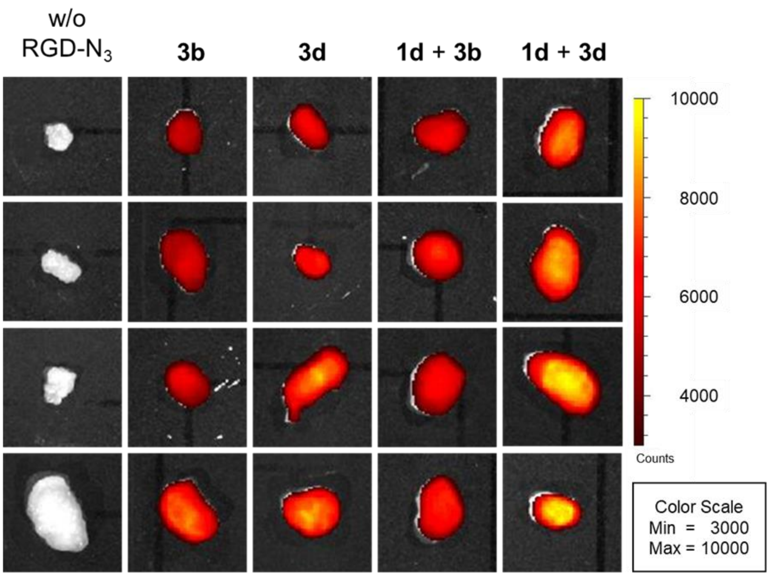

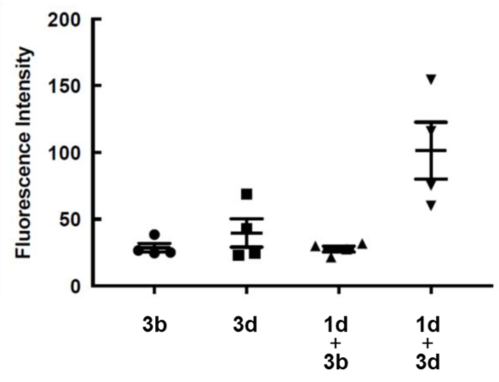

Glycan ligands	w/o RGD-N_3	1d
3b	28.7 ± 5.7	27.8 ± 3.9
3d	39.7 ± 18.4	101.4 ± 36.8

b) A549

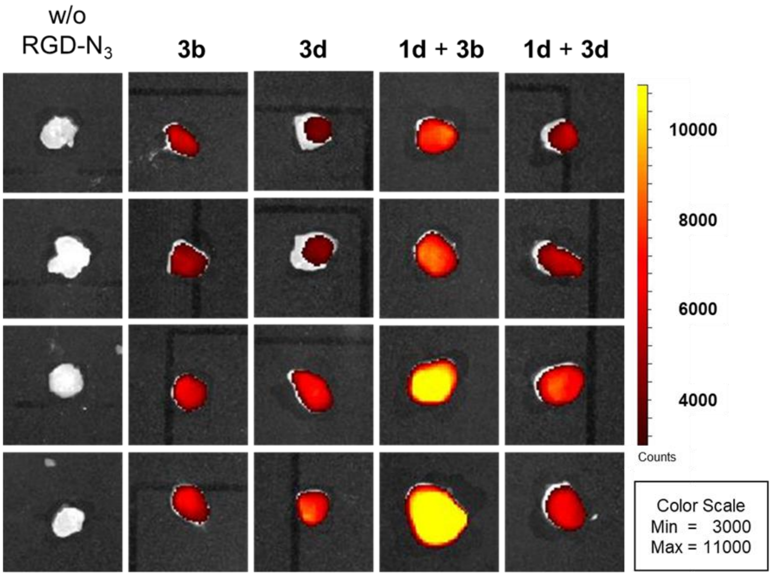

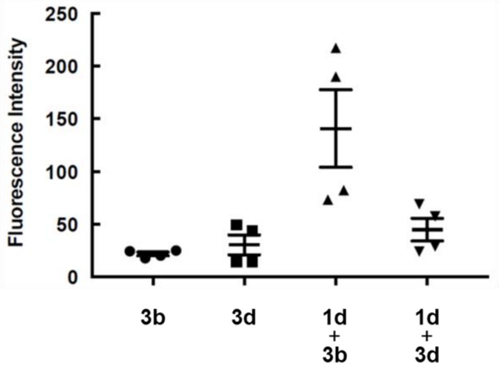

Glycan ligands	w/o RGD-N_3	1d
3b	21.9 ± 3.1	140.8 ± 63.7
3d	30.5 ± 16.3	44.8 ± 18.8

Supplementary Figure 18. Discrimination of a) HeLaS3 and b) A549 cancer tissues in xenografted mice. Data presented as mean ± SEM, *$p < 0.05$..

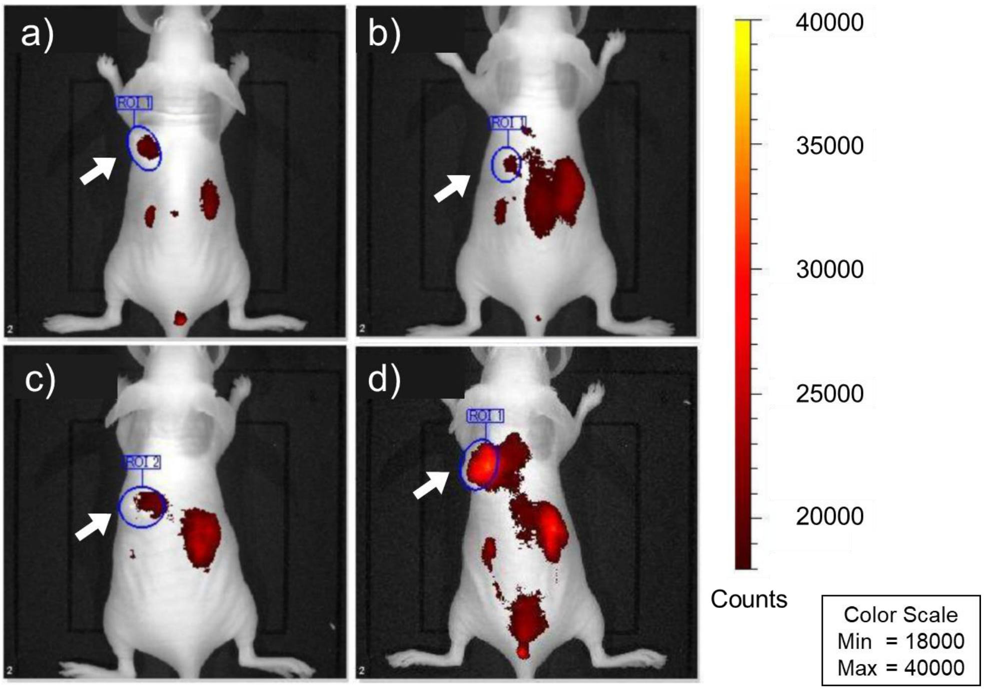

Supplementary Figure 19. Selective labeling of HeLaS3 cancer in xenografted mice. Mice were injected intravenously with a) **3b**, b) **3d**, c) **1d** then **3b**, and d) **1d** then **3d**. After 2.5 hours from second injection, the images were taken by a PerkinElmer IVIS Spectrum *in vivo* imaging system.

Supplementary Figue 20. NMR Spectra

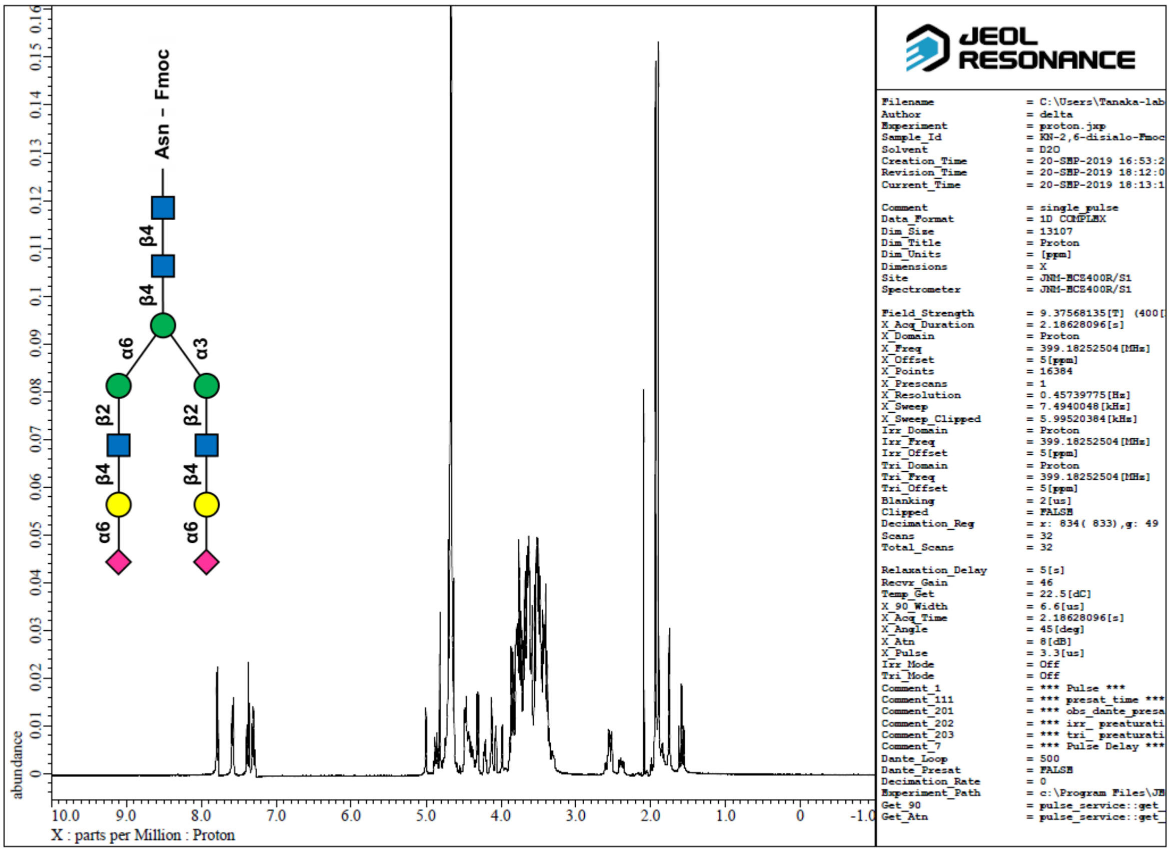

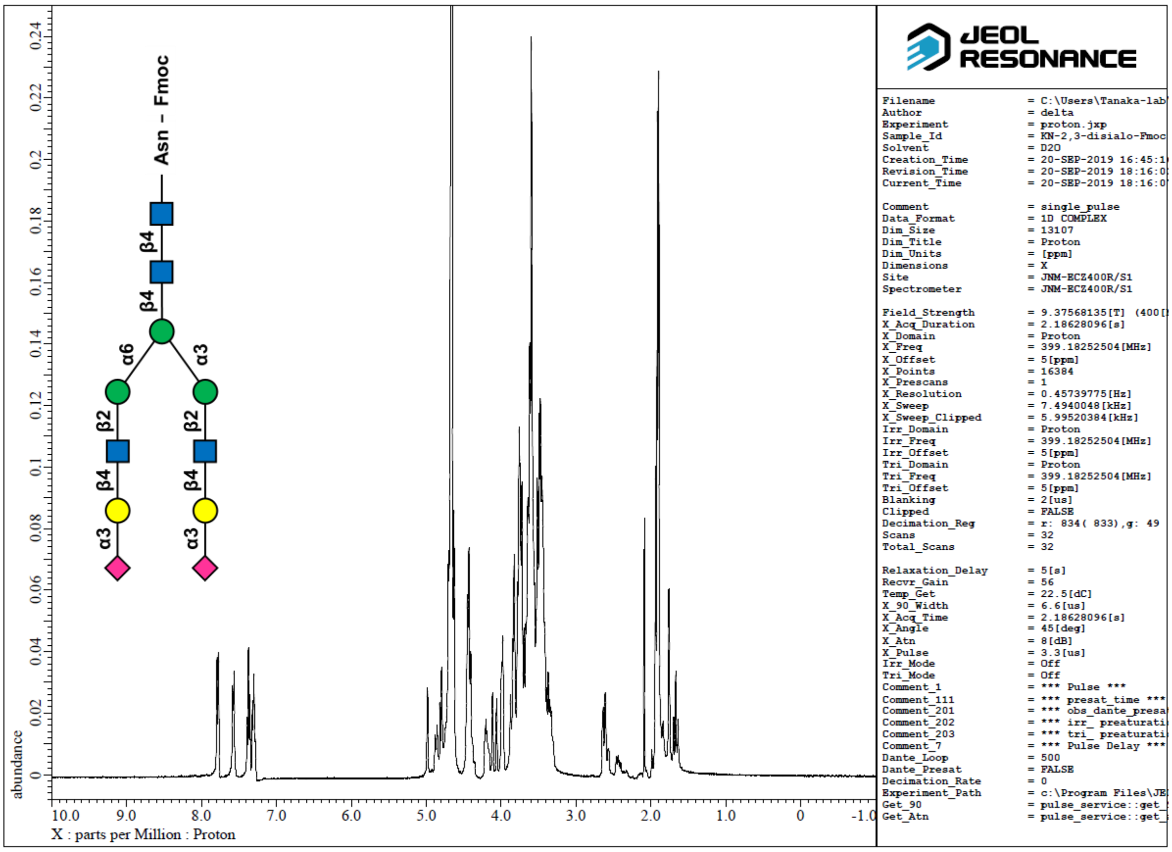

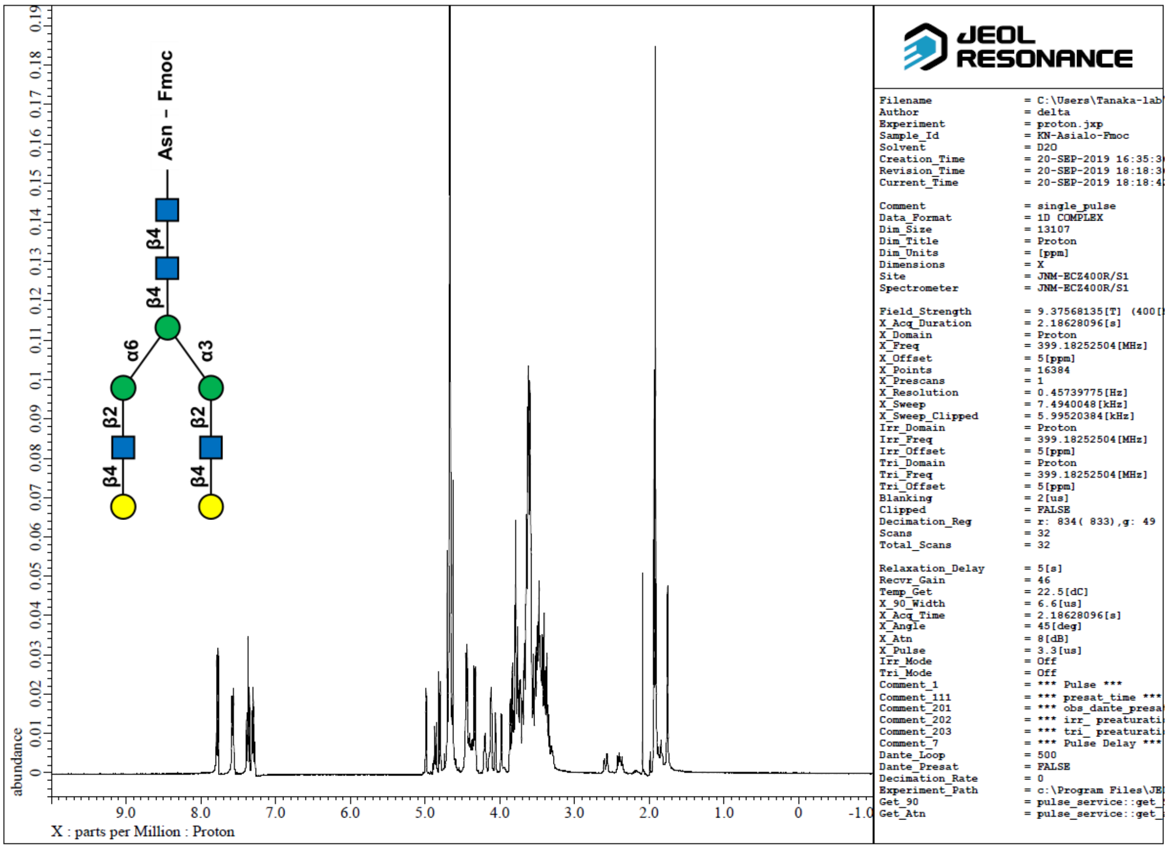

Asn - Fmoc
β4
β4
α6
α3
β2
β2
β4
β4
abundance
0.19
0.18
0.17
0.16
0.15
0.14
0.13
0.12
0.11
0.1
0.09
0.08
0.07
0.06
0.05
0.04
0.03
0.02
0.01
0
9.0 8.0 7.0 6.0 5.0 4.0 3.0 2.0 1.0 0 -1.0
X : parts per Million : Proton
JEOL RESONANCE
Filename = C:\Users\Tanaka-lab
Author = delta
Experiment = proton.jxp
Sample_Id = KN-Asialo-Fmoc
Solvent = D2O
Creation_Time = 20-SEP-2019 16:35:3
Revision_Time = 20-SEP-2019 18:18:3
Current_Time = 20-SEP-2019 18:18:4
Comment = single_pulse
Data_Format = 1D COMPLEX
Dim_Size = 13107
Dim_Title = Proton
Dim_Units = [ppm]
Dimensions = X
Site = JNM-ECZ400R/S1
Spectrometer = JNM-ECZ400R/S1
Field_Strength = 9.37568135[T] (400[
X_Acq_Duration = 2.18628096[s]
X_Domain = Proton
X_Freq = 399.18252504[MHz]
X_Offset = 5[ppm]
X_Points = 16384
X_Prescans = 1
X_Resolution = 0.45739775[Hz]
X_Sweep = 7.4940048[kHz]
X_Sweep_Clipped = 5.99520384[kHz]
Irr_Domain = Proton
Irr_Freq = 399.18252504[MHz]
Irr_Offset = 5[ppm]
Tri_Domain = Proton
Tri_Freq = 399.18252504[MHz]
Tri_Offset = 5[ppm]
Blanking = 2[us]
Clipped = FALSE
Decimation_Reg = r: 834(833),g: 49
Scans = 32
Total_Scans = 32
Relaxation_Delay = 5[s]
Recvr_Gain = 46
Temp_Get = 22.5[dC]
X_90_Width = 6.6[us]
X_Acq_Time = 2.18628096[s]
X_Angle = 45[deg]
X_Atn = 8[dB]
X_Pulse = 3.3[us]
Irr_Mode = Off
Tri_Mode = Off
Comment_1 = *** Pulse ***
Comment_111 = *** presat_time ***
Comment_201 = *** obs_dante_presa
Comment_202 = *** irr_ preaturati
Comment_203 = *** tri_ preaturati
Comment_7 = *** Pulse Delay ***
Dante_Loop = 500
Dante_Presat = FALSE
Decimation_Rate = 0
Experiment_Path = c:\Program Files\JE
Get_90 = pulse_service::get_
Get_Atn = pulse_service::get_

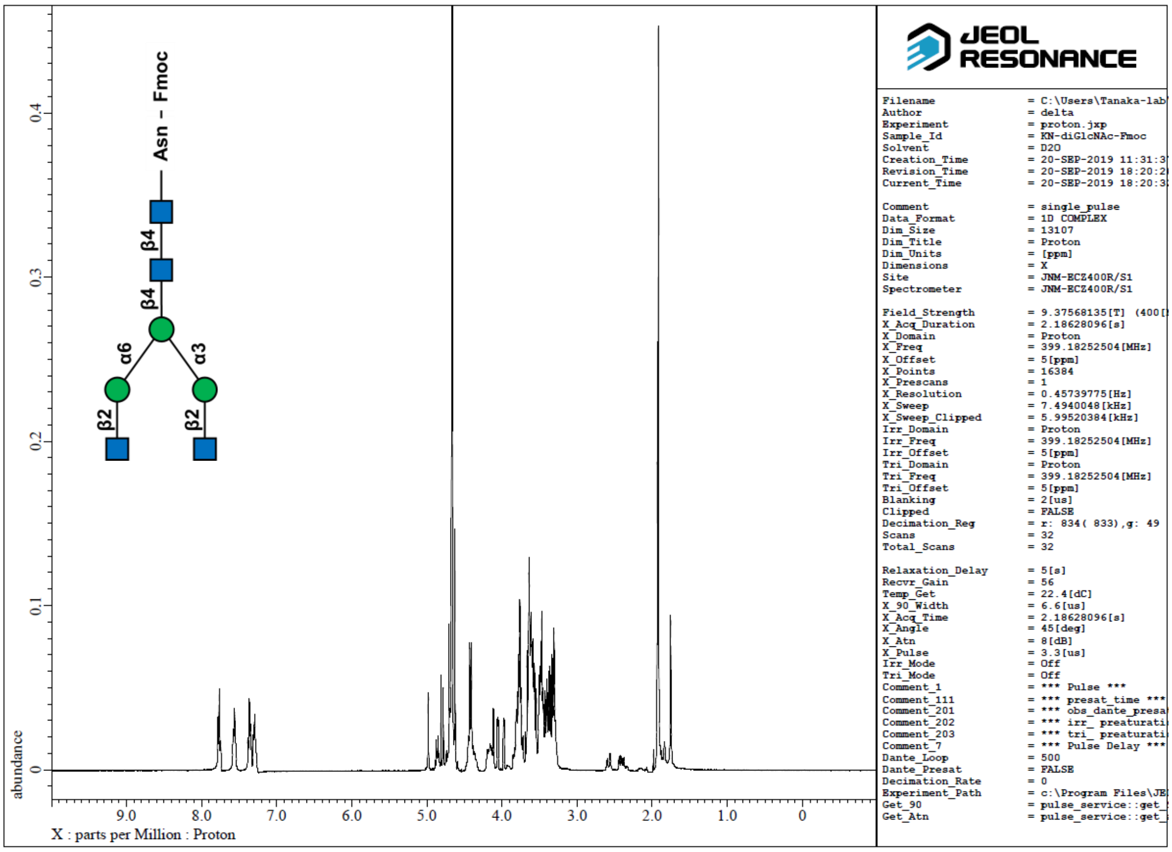

Asn - Fmoc
β4
β4
α6
α3
β2
β2
abundance
0.4
0.3
0.2
0.1
0
9.0 8.0 7.0 6.0 5.0 4.0 3.0 2.0 1.0 0
X : parts per Million : Proton
JEOL RESONANCE
Filename = C:\Users\Tanaka-lab
Author = delta
Experiment = proton.jxp
Sample_Id = KN-diGlcNAc-Fmoc
Solvent = D2O
Creation_Time = 20-SEP-2019 11:31:3
Revision_Time = 20-SEP-2019 18:20:2
Current_Time = 20-SEP-2019 18:20:3
Comment = single_pulse
Data_Format = 1D COMPLEX
Dim_Size = 13107
Dim_Title = Proton
Dim_Units = [ppm]
Dimensions = X
Site = JNM-ECZ400R/S1
Spectrometer = JNM-ECZ400R/S1
Field_Strength = 9.37568135[T] (400[
X_Acq_Duration = 2.18628096[s]
X_Domain = Proton
X_Freq = 399.18252504[MHz]
X_Offset = 5[ppm]
X_Points = 16384
X_Prescans = 1
X_Resolution = 0.45739775[Hz]
X_Sweep = 7.4940048[kHz]
X_Sweep_Clipped = 5.99520384[kHz]
Irr_Domain = Proton
Irr_Freq = 399.18252504[MHz]
Irr_Offset = 5[ppm]
Tri_Domain = Proton
Tri_Freq = 399.18252504[MHz]
Tri_Offset = 5[ppm]
Blanking = 2[us]
Clipped = FALSE
Decimation_Reg = r: 834(833),g: 49
Scans = 32
Total_Scans = 32
Relaxation_Delay = 5[s]
Recvr_Gain = 56
Temp_Get = 22.4[dC]
X_90_Width = 6.6[us]
X_Acq_Time = 2.18628096[s]
X_Angle = 45[deg]
X_Atn = 8[dB]
X_Pulse = 3.3[us]
Irr_Mode = Off
Tri_Mode = Off
Comment_1 = *** Pulse ***
Comment_111 = *** presat_time ***
Comment_201 = *** obs_dante_presa
Comment_202 = *** irr_ preaturati
Comment_203 = *** tri_ preaturati
Comment_7 = *** Pulse Delay ***
Dante_Loop = 500
Dante_Presat = FALSE
Decimation_Rate = 0
Experiment_Path = c:\Program Files\JE
Get_90 = pulse_service::get_
Get_Atn = pulse_service::get_

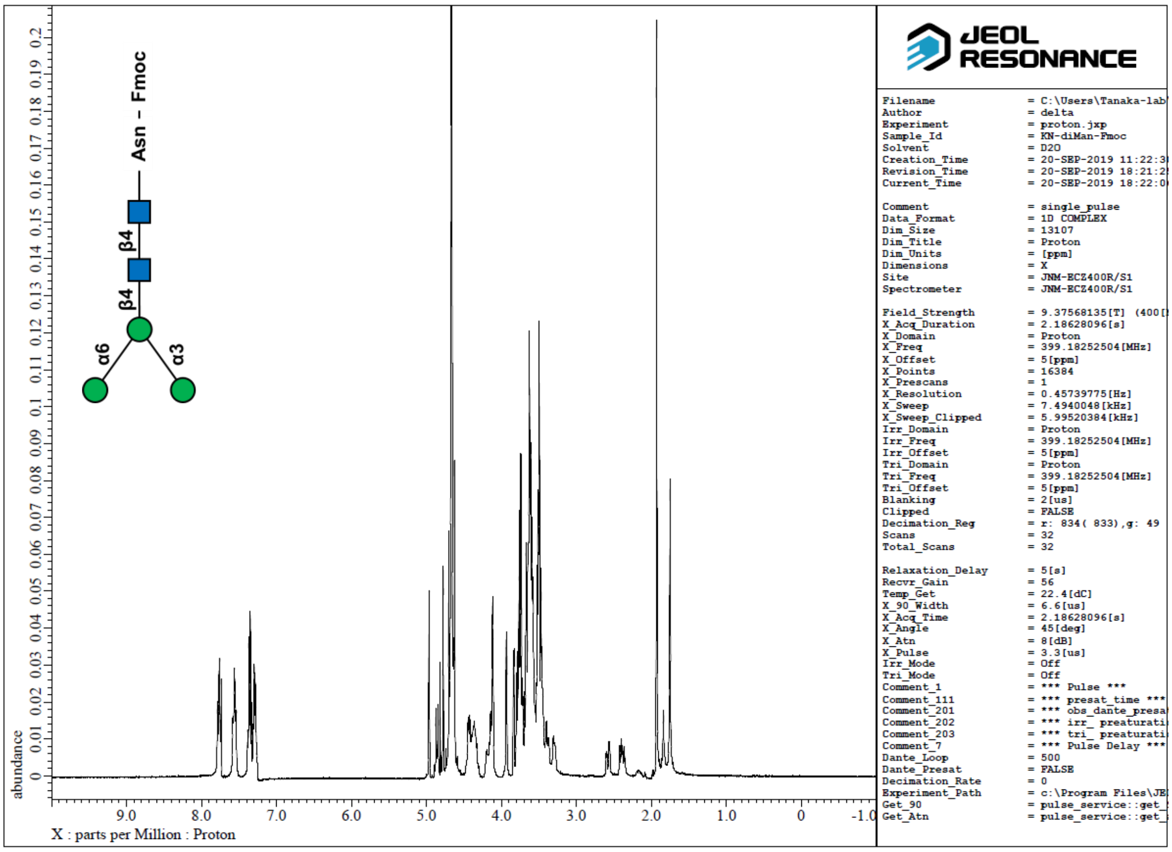
Asn - Fmoc
β4
β4
α6
α3
abundance
0 0.01 0.02 0.03 0.04 0.05 0.06 0.07 0.08 0.09 0.1 0.11 0.12 0.13 0.14 0.15 0.16 0.17 0.18 0.19 0.2
9.0 8.0 7.0 6.0 5.0 4.0 3.0 2.0 1.0 0 -1.0
X : parts per Million : Proton
JEOL RESONANCE
Filename = C:\Users\Tanaka-lab
Author = delta
Experiment = proton.jxp
Sample_Id = KN-diMan-Fmoc
Solvent = D2O
Creation_Time = 20-SEP-2019 11:22:3
Revision_Time = 20-SEP-2019 18:21:2
Current_Time = 20-SEP-2019 18:22:0
Comment = single_pulse
Data_Format = 1D COMPLEX
Dim_Size = 13107
Dim_Title = Proton
Dim_Units = [ppm]
Dimensions = X
Site = JNM-ECZ400R/S1
Spectrometer = JNM-ECZ400R/S1
Field_Strength = 9.37568135[T] (400[
X_Acq_Duration = 2.18628096[s]
X_Domain = Proton
X_Freq = 399.18252504[MHz]
X_Offset = 5[ppm]
X_Points = 16384
X_Prescans = 1
X_Resolution = 0.45739775[Hz]
X_Sweep = 7.4940048[kHz]
X_Sweep_Clipped = 5.99520384[kHz]
Irr_Domain = Proton
Irr_Freq = 399.18252504[MHz]
Irr_Offset = 5[ppm]
Tri_Domain = Proton
Tri_Freq = 399.18252504[MHz]
Tri_Offset = 5[ppm]
Blanking = 2[us]
Clipped = FALSE
Decimation_Reg = r: 834(833),g: 49
Scans = 32
Total_Scans = 32
Relaxation_Delay = 5[s]
Recvr_Gain = 56
Temp_Get = 22.4[dC]
X_90_Width = 6.6[us]
X_Acq_Time = 2.18628096[s]
X_Angle = 45[deg]
X_Atn = 8[dB]
X_Pulse = 3.3[us]
Irr_Mode = Off
Tri_Mode = Off
Comment_1 = *** Pulse ***
Comment_111 = *** presat_time ***
Comment_201 = *** obs_dante_presa
Comment_202 = *** irr_ preaturati
Comment_203 = *** tri_ preaturati
Comment_7 = *** Pulse Delay ***
Dante_Loop = 500
Dante_Presat = FALSE
Decimation_Rate = 0
Experiment_Path = c:\Program Files\JE
Get_90 = pulse_service::get_
Get_Atn = pulse_service::get_

Supplementary References

1. Kajihara, Y. et al. Prompt Chemoenzymatic Synthesis of Diverse Complex-Type Oligosaccharides and Its Application to the Solid-Phase Synthesis of a Glycopeptide with Asn-Linked Sialyl-undeca- and Asialo-nonasaccharides. *Chem. Eur. J.*, 10, 971-985 (2004).

2. Tanaka, K. et al. Noninvasive imaging of dendrimer-type N-glycan clusters: in vivo dynamics dependence on oligosaccharide structure. *Angew. Chem. Int. Ed.*, 49, 8195-8200 (2010).

3. Taichi, M. et al. In Situ Ligation of High- and Low-Affinity Ligands to Cell Surface Receptors Enables Highly Selective Recognition. *Adv. Sci.*, 1700147 (2017).
