# Supplementary material for: Cancer discrimination by on-cell N-glycan ligation
Source: Commun Chem. 2020 Feb 26;3:26. doi: 10.1038/s42004-020-0270-9 (PMC9814842; doi:10.1038/s42004-020-0270-9)
Supplement: Supplementary file 2 — Description of Additional Supplementary Files [file 42004_2020_270_MOESM2_ESM.pdf]

Description of Additional Supplementary Files

Supplementary Data 1. Data sets underlying Figures 3 and 4.
